# A Software Tool for Exploring the Relation between Diagnostic Accuracy and Measurement Uncertainty

**DOI:** 10.3390/diagnostics10090610

**Published:** 2020-08-19

**Authors:** Theodora Chatzimichail, Aristides T. Hatjimihail

**Affiliations:** Hellenic Complex Systems Laboratory, Kostis Palamas 21, 66131 Drama, Greece; tc@hcsl.com

**Keywords:** diagnostic accuracy measures, ROC curve, measurement uncertainty, diagnostic tests, screening tests, risk

## Abstract

Screening and diagnostic tests are used to classify people with and without a disease. Diagnostic accuracy measures are used to evaluate the correctness of a classification in clinical research and practice. Although this depends on the uncertainty of measurement, there has been limited research on their relation. The objective of this work was to develop an exploratory tool for the relation between diagnostic accuracy measures and measurement uncertainty, as diagnostic accuracy is fundamental to clinical decision-making, while measurement uncertainty is critical to quality and risk management in laboratory medicine. For this reason, a freely available interactive program was developed for calculating, optimizing, plotting and comparing various diagnostic accuracy measures and the corresponding risk of diagnostic or screening tests measuring a normally distributed measurand, applied at a single point in time in non-diseased and diseased populations. This is done for differing prevalence of the disease, mean and standard deviation of the measurand, diagnostic threshold, standard measurement uncertainty of the tests and expected loss. The application of the program is illustrated with a case study of glucose measurements in diabetic and non-diabetic populations. The program is user-friendly and can be used as an educational and research tool in medical decision-making.

## 1. Introduction

**Figure 1:**
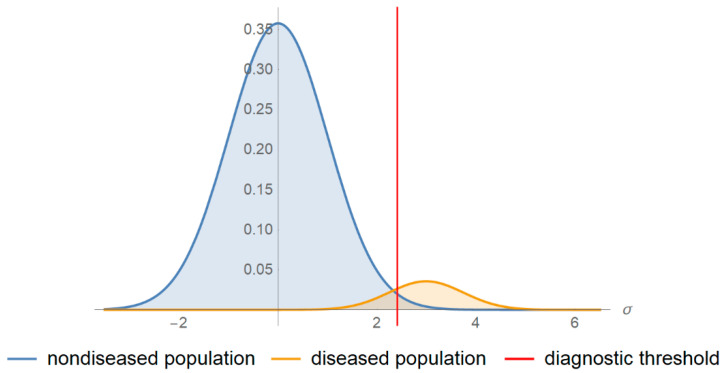
Probability density function plots. The probability density function plots of a measurand in a non-diseased and diseased population.

An increasing number of in vitro screening and diagnostic tests are extensively used as binary classifiers in medicine, to classify people into the non-overlapping classes of populations with and without a disease, which are categorized as quantitative and qualitative. The quantitative and many qualitative screening or diagnostic tests are based on measurements. There is a probability distribution of the measurements in each of the diseased and non-diseased populations. To classify the patients with and without a disease, using a test based on a measurement, a diagnostic threshold or cutoff point is defined. If the measurement is above the threshold, the patient is classified as test-positive; otherwise, the patient is classified as test-negative (Figure 1) or inversely. The possible test results are summarized in Table 1.

From the large number of diagnostic accuracy measures (DAM) appearing in the literature, only a few are used for evaluating the diagnostic accuracy in clinical research and practice [1]. These include the following:

1. Sensitivity (*Se)*, specificity (*Sp)*, diagnostic odds ratio (*DOR)*, likelihood ratio for positive or negative result (*LR* + and *LR −,* respectively), which are defined conditionally on the true disease status [2] and are prevalence invariant.
2. Overall diagnostic accuracy (*ODA)*, which is defined conditionally on the true disease status and is prevalence-dependent.
3. Positive predictive and negative predictive value (*PPV* and *NPV)*, which are defined conditionally on the test outcome and are prevalence-dependent.

The natural frequency and the equivalent probability definitions of the diagnostic accuracy measures derived from Table 1 and analyzed by the program are presented in Table 2. The symbols are explained in Appendix A.

Receiver operating characteristic (*ROC)* curves are also used for the evaluation of the diagnostic performance of a screening or diagnostic test [3]. *ROC* curves are plots of *Se* against 1-*Sp* of the test.

A related summary measure of diagnostic accuracy is the area under a *ROC* curve (*AUC)* [4,5]. The area over a *ROC* curve (*AOC)* has been proposed as a complementary summary measure of the diagnostic inaccuracy [6].

Recently, the predictive receiver operating characteristic (*PROC)* curves have also been proposed. *PROC* curves are plots of *PPV* against 1-*NPV* of the test [2].

For the optimization of binary classifiers, objective or loss functions have been proposed. They are based on diagnostic accuracy measures that can be maximized or minimized by finding the optimal diagnostic threshold. These measures include Youden’s index (*J*) [7], Euclidean distance of a *ROC* curve point from the point (0, 1) (*ED*) [8] and the concordance probability measure (*CZ)* [9]. The abovementioned measures are defined conditionally on the true disease status and are prevalence invariant. Their respective probability and natural frequency definitions are presented in Table 2.

The risk of a diagnostic or screening test is related to its diagnostic accuracy and is defined as its expected loss. Therefore, it depends upon the following (Table 2):

1. The expected loss for the testing procedure, for a true negative result, for a false negative result, for a true positive result and for a false positive result, defined on the same scale.
2. The probabilities for a true negative result, for a false negative result, for a true positive result and for a false positive result.

Risk is defined conditionally on the true disease status and is prevalence-dependent.

As there is inherent variability in any measurement process, there is measurement uncertainty, which is defined as a “parameter, associated with the result of a measurement, that characterizes the dispersion of the values that could reasonably be attributed to the measurand” [10]. The parameter may be the standard measurement uncertainty (*u*), expressed as a standard deviation and estimated as described in “Expression of Measurement Uncertainty in Laboratory Medicine” [11]. Bias may be considered as a component of the standard measurement uncertainty [12].

The measurement uncertainty is gradually replacing the total analytical error concept [13].

### Relation between Diagnostic Accuracy and Measurement Uncertainty

Although the estimation of measurement uncertainty is essential for quality assurance in laboratory medicine [11], its effect on clinical decision-making and consequently on clinical outcomes is rarely quantified [14]. As direct-outcome studies are very complex, a feasible first step is exploring the effect of measurement uncertainty on misclassification [15] and subsequently on diagnostic accuracy measures and the corresponding risk. Exploring this relation could assist the process of estimation of the optimal diagnostic threshold or the permissible measurement uncertainty.

## 2. Materials and Methods

For the calculation of the diagnostic accuracy measures, the following is assumed:

1. There is a reference (“gold standard”) diagnostic method classifying correctly a subject as diseased or non-diseased [16].
2. The parameters of the distributions of the measurand are known.
3. Either the values of the measurand or their transforms [17,18] are normally distributed in each of the diseased and non-diseased populations.
4. The measurement uncertainty is normally distributed and homoscedastic in the diagnostic threshold’s range.
5. If the measurement is above the threshold the patient is classified as test-positive otherwise as test-negative.

Hereafter, we use the term measurand to describe either the normally distributed value of a measurand or its normally distributed applicable transform.

Consequently, if *σ* is the standard deviation of the measurements of a screening or diagnostic test applied in a population (*P*)*, u* the standard measurement uncertainty and *σ_p_* the standard deviation of the measurand in the population, then we get the following equation:

(1) $$\sigma =\sqrt{\sigma_{P}^{2}+u^{2}}$$

The definitions of the diagnostic accuracy measures can be expressed in terms of sensitivity (*Se*) and specificity (*Sp*). These definitions are derived from Table 2 and presented in Table 3.

The functions of sensitivity (*Se)* and specificity (*Sp*), hence the functions of all the above diagnostic accuracy measures, can be expressed in terms of the cumulative distribution function of the normal distribution and therefore of the error function and the complementary error function.

The error function, *erf* (*x)*, is defined as follows:

(2) $$erf\left(x\right)=\frac{2}{\sqrt{\pi }}\int_{0}^{x}e^{-t^{2}}dt,\;x\geq 0$$

while the complementary error function, *erfc* (*x*), is defined as follows:

(3) $$erfc\left(x\right)=1-erf\left(x\right)=\frac{2}{\sqrt{\pi }}\int_{x}^{\infty }e^{-t^{2}}dt,\;x\geq 0$$

Following the definition of the sensitivity and specificity of a test (Table 2), the respective functions against diagnostic threshold (*d*) are calculated as follows:

(4) $$se\left(d,\mu_{D},\;\sigma_{D},u\right)=1-\Psi \left(d,\mu_{D},\sqrt{\sigma_{D}^{2}+u^{2}}\right)=\frac{1}{2}\left(1+erf\left(\frac{-d+\mu_{D}\;}{\sqrt{2\left(\sigma_{D}^{2}+u^{2}\right)}}\right)\right)$$

(5) $$sp\left(d,\mu_{\bar{D}},\;\sigma_{\bar{D}},u\right)=\Psi \left(d,\mu_{\bar{D}},\sqrt{\sigma_{\bar{D}}^{2}+u^{2}}\right)=\frac{1}{2}erfc\left(\frac{-d+\mu_{\bar{D}}\;}{\sqrt{2\left(\sigma_{\bar{D}}^{2}+u^{2}\right)}}\right)$$

where *Ψ* denotes the cumulative distribution function of a normal distribution; $\mu_{D}$ the mean and $\sigma_{D}$ the standard deviation of the measurand of the test in the diseased population; $\mu_{\bar{D}}$ the mean and $\sigma_{\bar{D}}$ the standard deviation of the measurand of the test in the non-diseased population; and *u* the standard measurement uncertainty of the test.

Then, the sensitivity function of a test against its specificity (*z*) is calculated as follows:

(6) $$se_{sp}\left(z,\mu_{D},\;\sigma_{D},\;\mu_{\bar{D}},\;\sigma_{\bar{D}},u\right)=1-\Psi \left(\Psi^{-1}\left(z,\mu_{\bar{D}},\sqrt{\sigma_{\bar{D}}^{2}+u^{2}}\right),\mu_{D},\sqrt{\sigma_{D}^{2}+u^{2}}\right)=\;\frac{1}{2}\left(1+erf\left(\frac{\mu_{D}-\mu_{\bar{D}}+\sqrt{2\left(\sigma_{\bar{D}}^{2}+u^{2}\right)}+erfc^{-1}\left(2z\;\right)}{\sqrt{2\left(\sigma_{D}^{2}+u^{2}\right)}}\right)\right),0\leq \;z\leq 1$$

The specificity function of a single test against its sensitivity (*y*) is calculated as follows:

(7) $$sp_{se}\left(y,\mu_{D},\;\sigma_{D},\;\mu_{\bar{D}},\;\sigma_{\bar{D}},u\right)=\Psi \left(\Psi^{-1}\left(1-y,\mu_{\bar{D}},\sqrt{\sigma_{\bar{D}}^{2}+u^{2}}\right),\mu_{\bar{D}},\sqrt{\sigma_{\bar{D}}^{2}+u^{2}}\right)\;=\frac{1}{2}erfc\left(\frac{-\mu_{D}+\mu_{\bar{D}}+\sqrt{2\left(\sigma_{D}^{2}+u^{2}\right)}erfc^{-1}\left(2-2y\right)}{\sqrt{2\left(\sigma_{\bar{D}}^{2}+u^{2}\right)}}\right),0\leq \;y\leq 1$$

Following Table 3 and Equations (4)–(7), the diagnostic accuracy measures of a test are defined as functions of either its diagnostic threshold, sensitivity, or specificity. Consequently, the derived parametric equations defining each measure can be used to explore the relations between any two measures.

Following the definition of the *ROC* curves and assuming a normal probability density function of the measurands of each of the diseased and non-diseased populations, the *ROC* function is calculated as follows:

(8) $$roc\left(t,\;\mu_{\bar{D}},\;\mu_{D},\sigma_{\bar{D,}},\;\sigma_{D},u\right)=S\left(S^{-1}\left(t,\;\mu_{\bar{D}},\sqrt{\;\sigma_{\bar{D}}^{2}+u^{2}}\right),\;\mu_{D},\;\sqrt{\;\sigma_{D}^{2}+u^{2}}\right),0\leq \;t\leq 1$$

where *S* denotes the survival function of normal distribution.

Consequently, we get the following:

(9) $$roc\left(t,\;\mu_{\bar{D}},\;\mu_{D},\sigma_{\bar{D}},\;\sigma_{D},u\right)=\frac{1}{2}erfc\left(\frac{-\mu_{D}+\mu_{\bar{D}}+\sqrt{2\left(\sigma_{\bar{D}}^{2}+u^{2}\right)erfc^{-1}}\left(2t\right)}{\sqrt{2\left(\sigma_{D}^{2}+u^{2}\right)}}\right),0\leq \;t\leq 1$$

The function of the area under the *ROC* curve is defined as follows:

(10) $$auc\left(\;\mu_{\bar{D}},\;\mu_{D},\sigma_{\bar{D,}},\;\sigma_{D},u\right)=\int_{0}^{1}roc\left(t,\;\mu_{\bar{D}},\;\mu_{D},\sigma_{\bar{D}},\;\sigma_{D},u\right)dt$$

Moreover, it is calculated as follows:

(11) $$auc\left(\;\mu_{\bar{D}},\;\mu_{D},\sigma_{\bar{D,}},\;\sigma_{D},u\right)=\Phi \left(\frac{\mu_{D}-\;\mu_{\bar{D}}}{\sqrt{\sigma_{\bar{D}}^{2}+\;\sigma_{D}^{2}+2u^{2}}}\right)$$

where *Φ* denotes the cumulative distribution function of the standard normal distribution.

The function of the area over the *ROC* curve is defined as follows:

(12) $$aoc\left(\mu_{\bar{D}},\;\mu_{D},\sigma_{\bar{D,}},\;\sigma_{D},u\right)=1-auc\left(\mu_{\bar{D}},\;\mu_{D},\sigma_{\bar{D,}},\;\sigma_{D},u\right)$$

Another *ROC* curve related quantity is the Euclidean distance (*ED)* of a *ROC* curve point $\left(t,roc\left(t,\;\mu_{\bar{D}},\;\mu_{D},\sigma_{\bar{D}},\;\sigma_{D},u\right)\right)$ from the point (0, 1) or equivalently the Euclidean distance of the point (*Se*, *Sp*) from the point (1, 1) of perfect diagnostic accuracy. The respective function is defined as follows:

(13) $$ed\left(t,\;\mu_{\bar{D}},\;\mu_{D},\sigma_{\bar{D}},\;\sigma_{D},u\right)=\sqrt{t^{2}+{\left(1-roc\left(t,\;\mu_{\bar{D}},\;\mu_{D},\sigma_{\bar{D}},\;\sigma_{D},u\right)\right)}^{2}}$$

The predictive *ROC* (*PROC*) curve relation is defined as follows [2]:

(14) $$proc\left(t,\;\mu_{\bar{D}},\;\mu_{D},\sigma_{\bar{D,}},\;\sigma_{D},u\right)=ppv\left(npv^{-1}\left(1-t,\;\mu_{\bar{D}},\;\mu_{D},\sigma_{\bar{D,}},\;\sigma_{D},u\right),\;\mu_{\bar{D}},\;\mu_{D},\sigma_{\bar{D,}},\;\sigma_{D},u\right)$$

This relation cannot be expressed in terms of elementary or survival functions.

**Figure 2:**
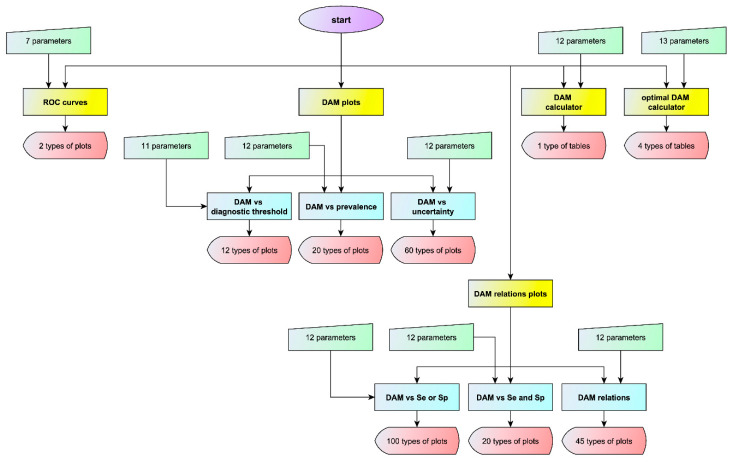
Program flowchart. The flowchart of the program with the number of input parameters and of output types for each module or submodule (DAM: diagnostic accuracy measure).

To explore the relation between diagnostic accuracy measures or the corresponding risk and measurement uncertainty, an interactive program written in Wolfram Language [19] was developed in Wolfram Mathematica^®^, ver. 12.1 [20]. This program was designed to provide five modules and six submodules for calculating, optimizing, plotting and comparing various diagnostic accuracy measures and the corresponding risk of two screening or diagnostic tests, applied at a single point in time in non-diseased and diseased populations (Figure 2). The two tests measure the same measurand, for varying values of the prevalence of the disease, the mean and standard deviation of the measurand in the populations and the standard measurement uncertainty of the tests. The two tests differ in measurement uncertainty. It is assumed that the measurands and the measurement uncertainty are normally distributed.

Parts of this program have been presented in a series of demonstrations, at Wolfram Demonstration Project of Wolfram Research [6,21,22,23,24,25,26,27].

The program is freely available as a Wolfram Mathematica^®^ notebook (.nb) at: https://www.hcsl.com/Tools/Relation.nb. It can be run on Wolfram Player^®^ or Wolfram Mathematica^®^ (see Appendix B). Detailed description of the interface of the program is available as Supplementary Material.

## 3. Results

### 3.1. Interface of the Program

The modules and the submodules of the program include panels with controls which allow the interactive manipulation of various parameters, as described in detail in Supplementary Material. These are the following:

#### 3.1.1. ROC Curves Module

The receiver operating characteristic (*ROC*) curves or the predictive receiver operating characteristic (*PROC*) curves of the two tests are plotted.

**Figure 3:**
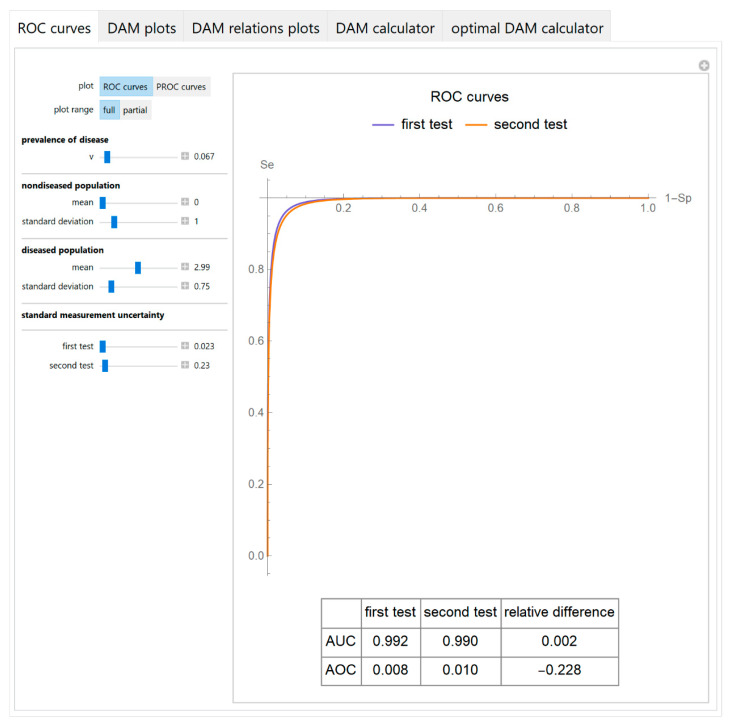
*ROC* curves module screenshot. *ROC* curves plots of two screening or diagnostic tests measuring the same measurand with different uncertainties, with the settings at the left.

A table with the respective *AUC* and *AOC* and their relative difference is also presented with the *ROC* curves plot (Figure 3).

#### 3.1.2. Diagnostic Accuracy Measures Plots Module

It includes the following submodules:

- Diagnostic accuracy measures against diagnostic threshold

**Figure 4:**
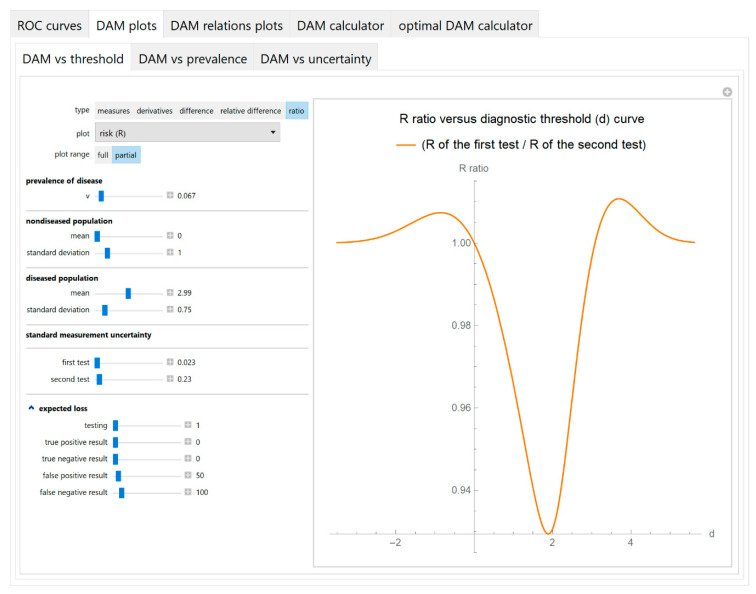
DAM plots module, DAM against threshold submodule screenshot. Ratio of the risk (*R*) of two screening or diagnostic tests measuring the same measurand with different uncertainties, against diagnostic threshold (*d*) curve plot, with the settings at the left.

The values of the diagnostic accuracy measures or the corresponding risk of the two tests, their partial derivatives with respect to standard measurement uncertainty, their difference, relative difference and ratio are plotted against the diagnostic threshold of each test (Figure 4).

- Diagnostic accuracy measures against prevalence

**Figure 5:**
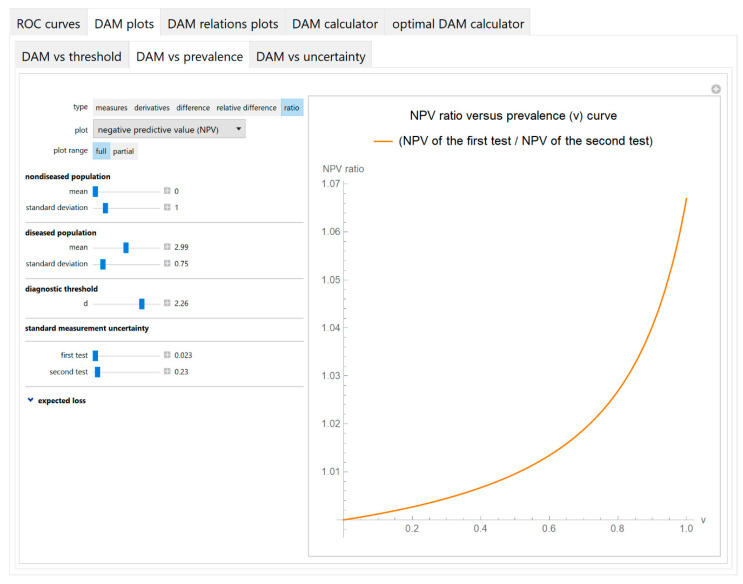
DAM plots module, DAM against prevalence submodule screenshot. Ratio of the negative predictive value (*NPV*) of the two screening or diagnostic tests measuring the same measurand with different uncertainties, against prevalence (*v*) of the disease curve plot, with the settings at the left.

The values of the diagnostic accuracy measures or the corresponding risk of the two tests, their partial derivatives with respect to standard measurement uncertainty, their difference, relative difference and ratio are plotted against the prevalence of the disease (Figure 5).

- Diagnostic accuracy measures against standard measurement uncertainty

**Figure 6:**
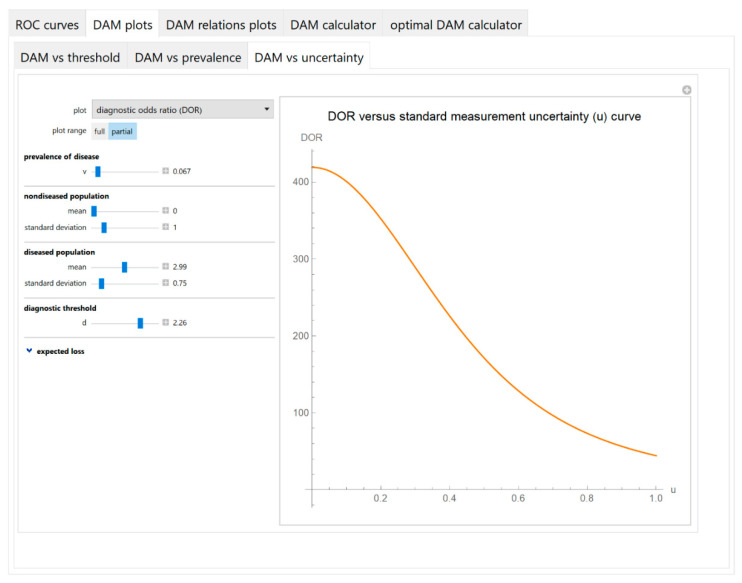
DAM plots module, DAM against uncertainty submodule screenshot. Diagnostic odds ratio (*DOR*) against standard measurement uncertainty (*u*) curve plot with the settings shown at the left.

The values of the diagnostic accuracy measures or the corresponding risk of a test are plotted against the standard measurement uncertainty of the test (Figure 6).

#### 3.1.3. Diagnostic Accuracy Measures Relations Plots Module

It includes the following submodules:

- Diagnostic accuracy measures against sensitivity or specificity

**Figure 7:**
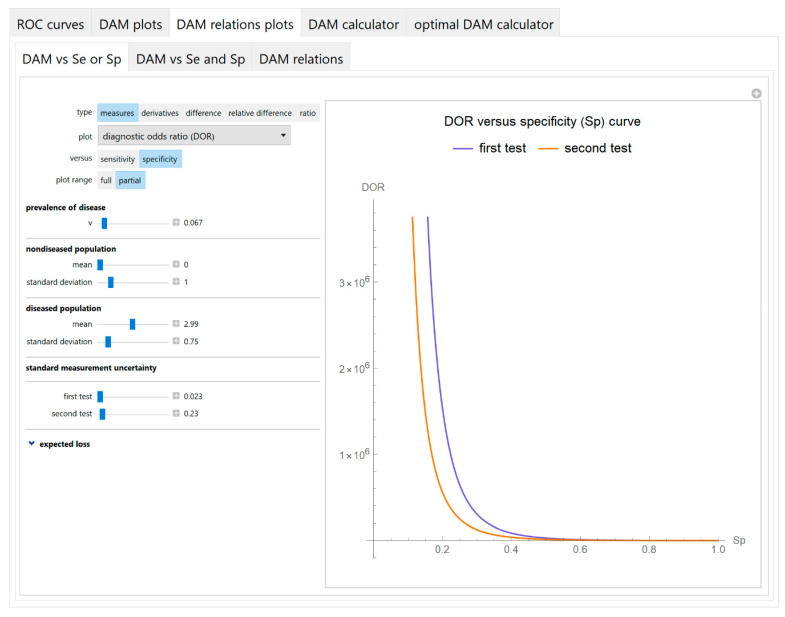
DAM relations plots module, DAM against sensitivity or specificity submodule screenshot. Diagnostic odds ratio (*DOR*) of two screening or diagnostic tests measuring the same measurand with different uncertainties, against specificity (*Sp*) curve plot, with the settings shown at the left.

The values of the diagnostic accuracy measures or the corresponding risk of the two tests, their partial derivatives with respect to standard measurement uncertainty, their difference, relative difference and ratio are plotted against either the sensitivity or the specificity of each test (Figure 7).

- Diagnostic accuracy measures against sensitivity and specificity

**Figure 8:**
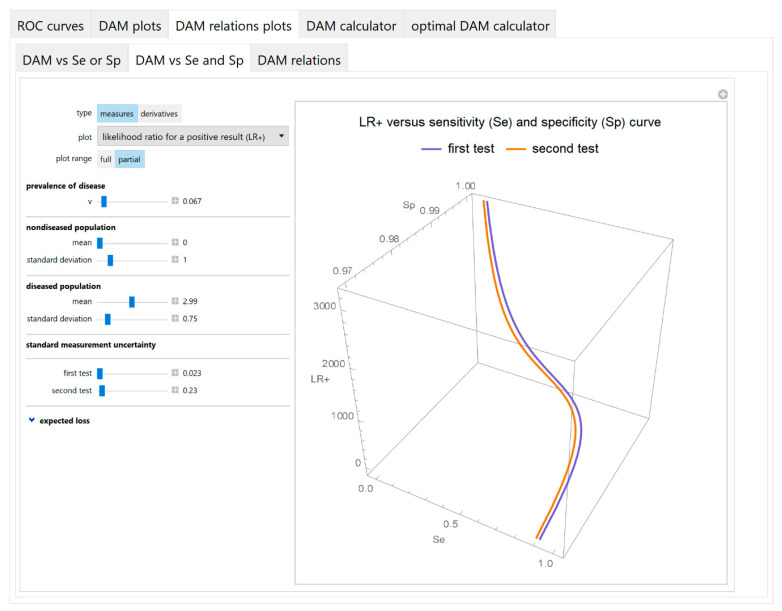
DAM relations plots module, DAM against sensitivity and specificity submodule screenshot. Likelihood ratio for a positive test result (LR +) of two screening or diagnostic tests measuring the same measurand with different uncertainties, against sensitivity (*Se*) and specificity (*Sp*) curves plot, with the settings shown at the left.

The values of the diagnostic accuracy measures or the corresponding risk of the two tests or their partial derivatives, with respect to standard measurement uncertainty, are plotted against the sensitivity and the specificity of each test in three-dimensional line plots (Figure 8).

- Diagnostic accuracy measures relations

**Figure 9:**
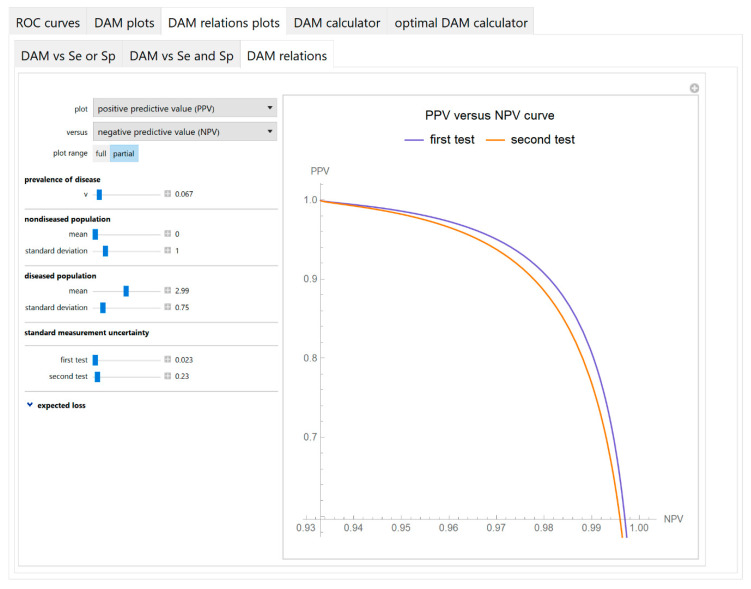
DAM relations plots module, DAM relations submodule screenshot. Positive predictive value (*PPV*) of two screening or diagnostic tests measuring the same measurand with different uncertainties, against negative predictive value (*NPV*) curves plot, with the settings at the left.

As any two of the diagnostic accuracy measures can be expressed as functions of their sensitivities, their respective parametric equations are plotted to show the relations between the values of the two measures of each test (Figure 9).

#### 3.1.4. Diagnostic Accuracy Measures Calculator Module

**Figure 10:**
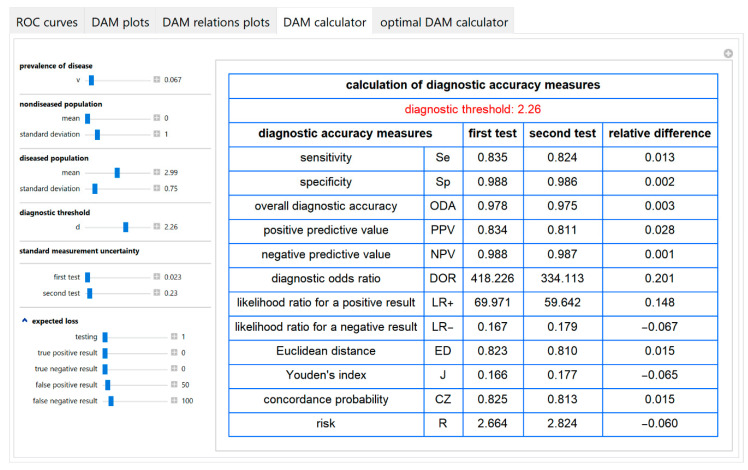
DAM calculator module screenshot. Calculated diagnostic accuracy measures of two screening or diagnostic tests measuring the same measurand with different uncertainties and their relative differences, with the settings at the left.

The values of various diagnostic accuracy measures and the corresponding risk of each of the two tests and their respective relative differences, at a selected diagnostic threshold, are calculated and presented in a table (Figure 10).

#### 3.1.5. Optimal Diagnostic Accuracy Measures Calculator Module

**Figure 11:**
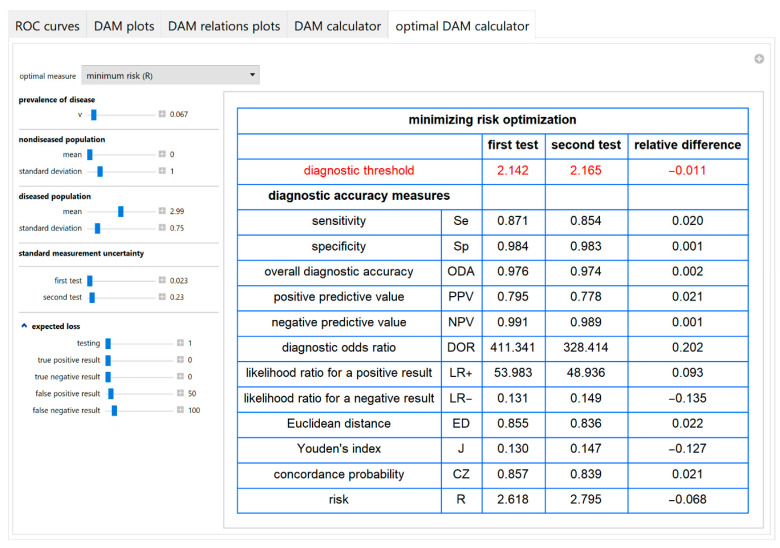
Optimal DAM calculator module screenshot. Calculated diagnostic accuracy measures of two screening or diagnostic tests measuring the same measurand with different uncertainties, minimizing risk (*R*) and their relative differences, with the settings at the left.

An optimal diagnostic threshold for each test is calculated according to a selected objective or loss function. Then the values of various diagnostic accuracy measures and the corresponding risk of each of the two tests, at the respective optimal threshold, are presented in a table (Figure 11).

### 3.2. Illustrative Case Study

The program was applied to a bimodal joint distribution, based on log-transformed blood glucose measurements in non-diabetic and diabetic Malay populations, during an oral glucose tolerance test (OGTT) [28]. Briefly, after the ingestion of 75 g glucose monohydrate, the two-hour postprandial blood glucose of 2667 Malay adults, aged 40–49 years, was measured with reflectance photometry. To apply the program, it was assumed that the prevalence of diabetes was 0.067, the measurement coefficient of variation and bias were equal to 4% and 2%, respectively and the log-transformed measurands of each population were normally distributed, as shown in Figure 1. The normalized log-transformed measurand means and standard deviations in the diseased and non-diseased populations, the standard measurement uncertainty and the diagnostic threshold were expressed in units equal to the standard deviation of the log-transformed measurand in the non-diseased population. The normalized log-transformed diagnostic threshold 2.26 corresponds to the American Diabetes Association (ADA) diagnostic threshold for diabetes of the two-hour postprandial glucose during OGTT that is equal to 11.1 mmol/L [29]. The normalized log-transformed standard measurement uncertainties 0.023 and 0.23 correspond to standard measurement uncertainties equal to 1% and 10% of the mean of the measurand of the non-diabetic population or equivalently to a coefficient of variation equal to 1% and 10%, respectively.

The parameter settings of the illustrative case study are presented in Table 4. The results of the application of the program are presented:

1. In the plots of Figure 3, Figure 4, Figure 5, Figure 6, Figure 7, Figure 8 and Figure 9, Figure 12, Figure 13, Figure 14, Figure 15, Figure 16 and Figure 17.
2. In the tables of Figure 10 and Figure 11.
3. In Table 5.

In this case, the measurement uncertainty has relatively little effect on the *ROC* and *PROC* curves, on *AUC*, sensitivity, specificity, overall diagnostic accuracy, positive predictive value, negative predictive value, Euclidean distance and concordance probability of the test, in accordance with previous findings [30,31]. Measurement uncertainty has a relatively greater effect on diagnostic odds ratio, on likelihood ratio for a positive or negative result, Youden’s index and risk.

As a result, the measurement uncertainty has relatively little effect on the optimal diagnostic thresholds maximizing the Youden’s index or the concordance probability or minimizing the Euclidean distance. Conversely, it has a relatively greater effect on the optimal diagnostic thresholds minimizing risk (Table 5).

## 4. Discussion

The purpose of this program is to explore the relation between diagnostic accuracy measures and measurement uncertainty, as diagnostic accuracy is fundamental to clinical decision-making, while defining the permissible measurement uncertainty is critical to quality and risk management in laboratory medicine. The current pandemic of the novel corona virus disease 2019 (COVID-19) has demonstrated these convincingly [32,33,34,35,36,37].

There has been extensive research on either diagnostic accuracy or measurement uncertainty; however, such research is very limited on both subjects [14,38,39].

**Figure 12:**
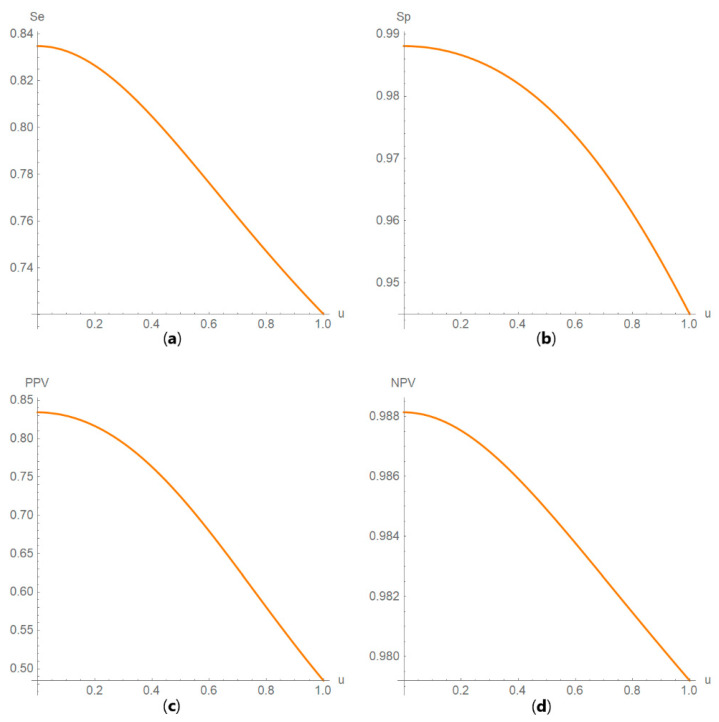
DAM against uncertainty plots. Plots of (**a**) sensitivity (*Se*), (**b**) specificity (*Sp*), (**c**) positive predictive value (*PPV*) and (**d**) negative predictive value (*NPV*) against standard measurement uncertainty (*u*) curves, with the respective parameters in Table 4.

**Figure 13:**
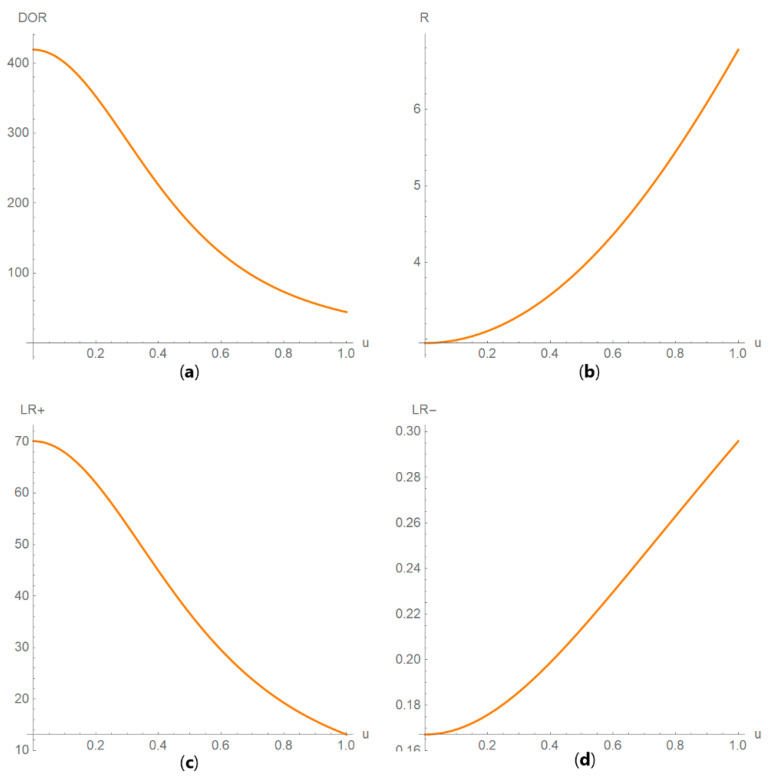
DAM against uncertainty plots. Plots of (**a**) diagnostic odds ratio (*DOR*), (**b**) risk (*R*), (**c**) likelihood ratio for a positive result (*LR* +) and (**d**) likelihood ratio for a negative result (*LR* −) against standard measurement uncertainty (*u*) curves, with the respective parameters in Table 4.

**Figure 14:**
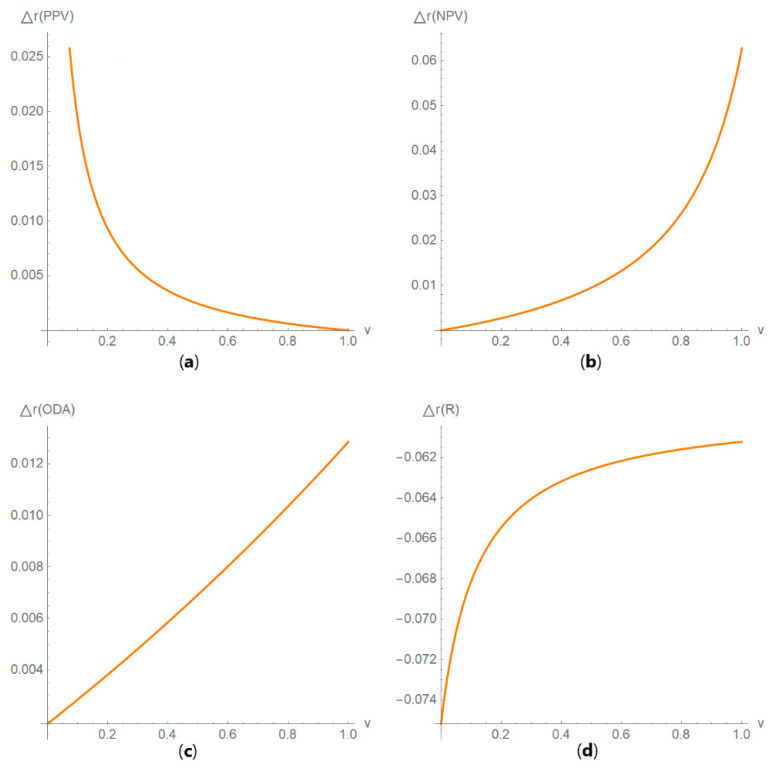
DAM relative differences against prevalence plots. Plots of the relative difference of the (**a**) positive predictive value (*PPV*), (**b**) negative predictive value (*NPV*), (**c**) overall diagnostic accuracy (*ODA*) and (**d**) risk (*R*) of two diagnostic or screening tests measuring the same measurand with different uncertainties, against prevalence (*v*) curves, with the respective parameters in Table 4.

**Figure 15:**
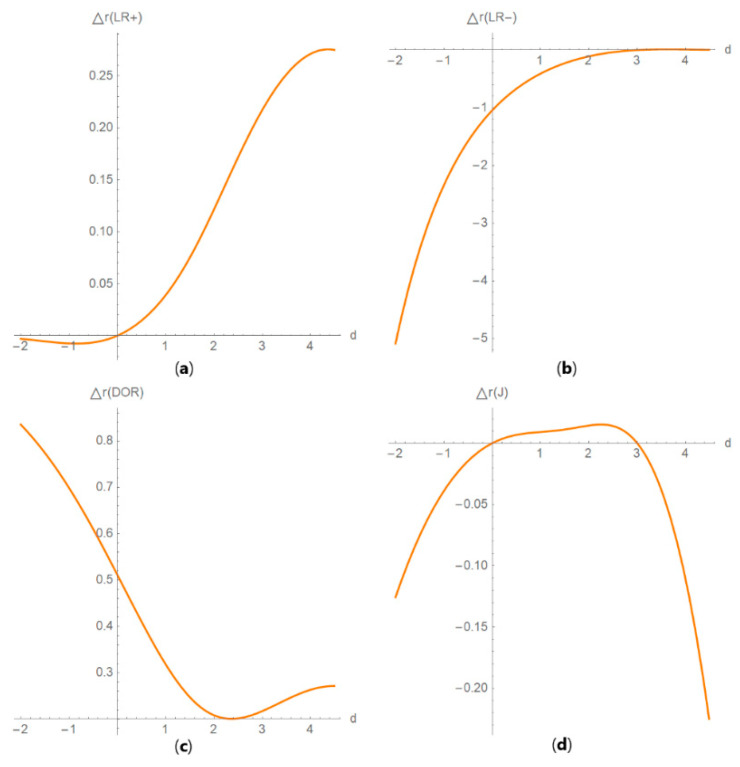
DAM relative differences against diagnostic threshold plots. Plots of the relative difference of the (**a**) likelihood ratio for a positive result (*LR* +), (**b**) likelihood ratio for a negative result (*LR* −), (**c**) diagnostic odds ratio (DOR) and (**d**) Youden’s index (*J*) of two screening or diagnostic tests measuring the same measurand with different uncertainties, against diagnostic threshold (*d*) curves, with the respective parameters in Table 4.

**Figure 16:**
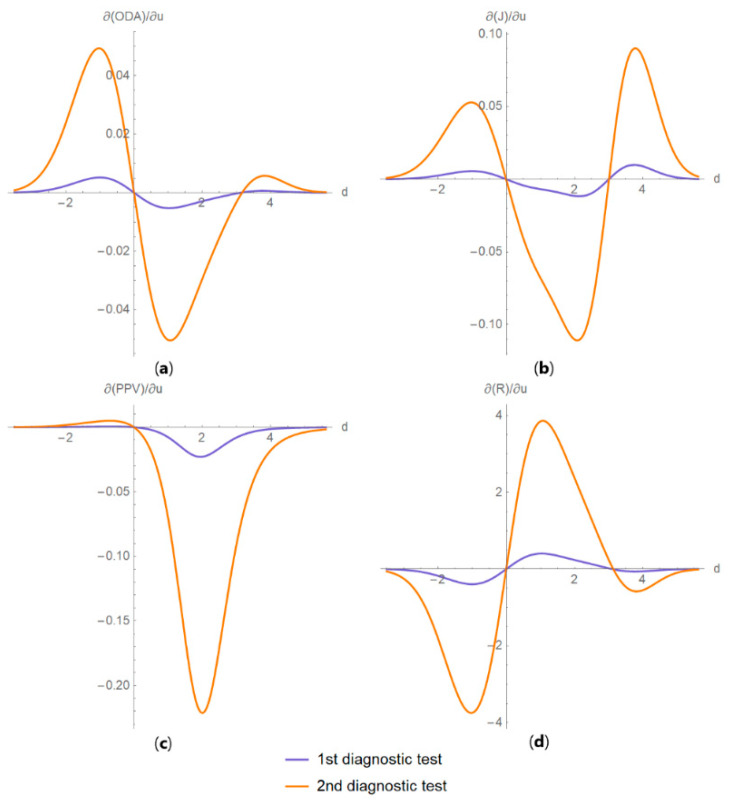
DAM partial derivatives against diagnostic threshold plots. Plots of partial derivatives of (**a**) overall diagnostic accuracy (*ODA*), (**b**) Youden’s index (*J*), (**c**) positive predictive value (*PPV*) and (**d**) risk (*R*), with respect to measurement uncertainty, of two diagnostic or screening tests measuring the same measurand with different uncertainties, against diagnostic threshold (*d*) curves, with the parameters in Table 4.

**Figure 17:**
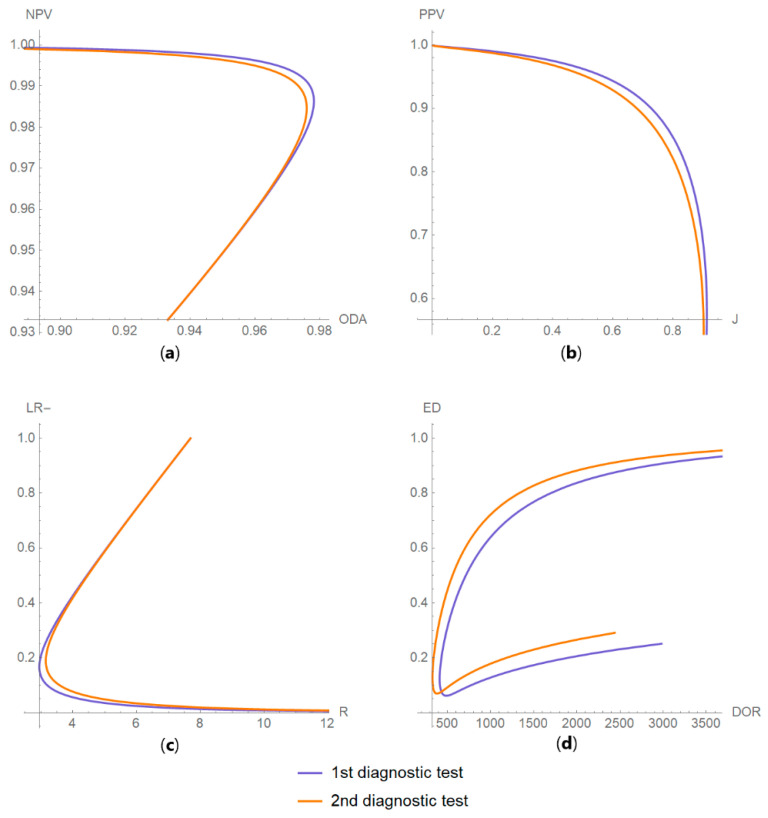
DAM relations plots. Plots of the relations between (**a**) negative predictive value (*NPV*) and overall diagnostic accuracy (*ODA*); (**b**) positive predictive value (*PPV*) and Youden’s index (*J*); (**c**) likelihood ratio for a negative result (*LR* −) and risk (*R*); and (**d**) Euclidean distance (*ED*) and diagnostic odds ratio (*DOR*), of two diagnostic or screening tests measuring the same measurand with different uncertainties, with the respective parameters in Table 4.

This program demonstrates the relation between the diagnostic accuracy measures and the measurement uncertainty for screening or diagnostic tests measuring a single measurand (Figure 3, Figure 4, Figure 5, Figure 6, Figure 7, Figure 8, Figure 9, Figure 10, Figure 11, Figure 12, Figure 13, Figure 14, Figure 15, Figure 16 and Figure 17). This relation depends on the population parameters, including the prevalence of the disease (Figure 5 and Figure 14) and on the diagnostic threshold (Figure 4, Figure 15 and Figure 16). In addition, measurement uncertainty affects the relation between any two of the diagnostic accuracy measures (Figure 7, Figure 8, Figure 9 and Figure 17).

As the program provides plots of the partial derivative of the diagnostic accuracy measures with respect to the standard measurement uncertainty, it offers a more detailed insight (Figure 16). In antithesis to the complexity of the relation, the program simplifies its exploration with a user-friendly interface.

Furthermore, it provides calculators for the calculation of the effects of measurement uncertainty on the diagnostic accuracy measures and corresponding risk (Figure 10) and for calculating the diagnostic threshold optimizing the objective and loss functions of Section 1 (Figure 11).

The counterintuitive finding that the measurement uncertainty has relatively little effect on the *ROC* and *PROC* curves, on *AUC*, sensitivity, specificity, overall diagnostic accuracy, positive predictive value, negative predictive value, Euclidean distance and concordance probability suggests that we should reconsider their interpretation in medical decision-making. However, further research is needed to explore the effect of measurement uncertainty on diagnostic accuracy measures with different clinically and laboratory relevant parameter settings. Furthermore, clinical laboratories should consider including measurement uncertainty in each test result report.

Compared to the risk measure, a shortcoming of Youden’s index, Euclidean distance of a *ROC* curve point from the point (0, 1) and concordance probability as objective functions is that they do not differentiate the relative significance of a true negative and a true positive test result or equivalently of a false-negative and a false-positive test result. Accordingly, in the case study, the optimal diagnostic thresholds maximizing the Youden’s index or the concordance probability or minimizing the Euclidean distance are considerably less than the ADA diagnostic threshold for diabetes of the two-hour postprandial glucose during OGTT (Table 5). Nevertheless, the optimal diagnostic threshold minimizing the risk can be close to the ADA threshold, with specific expected loss settings (Figure 11). Although risk assessment is evolving as the preferred method for optimization of medical decision-making [40] and for quality assurance in laboratory medicine [41], the estimation of expected loss for each test result (Table 2 and Table 3) is still a complex task. In the future, as the potential of the data analysis will increase exponentially, expected loss could be estimated by using evidence-based methods.

Shortcomings of this program are the following assumptions used for the calculations:

1. The existence of a “gold standard” diagnostic method. If a “gold standard” does not exist, there are alternative approaches for the estimation of diagnostic accuracy measures [42].
2. The parameters of the distributions of the measurand are assumed to be known. In practice, they are estimated [43].
3. The normality of either the measurements or their applicable transforms [17,18,44,45]; however, this is usually valid. There is related literature on the distribution of measurements of diagnostic tests, in the context of reference intervals and diagnostic thresholds or clinical decision limits [46,47,48,49,50].
4. The bimodality of the measurands that is generally accepted, although unimodal distributions could be considered [51,52].
5. The measurement uncertainty homoscedasticity in the diagnostic thresholds range. If measurement uncertainty is heteroscedastic, thus skewing the measurement distribution, appropriate transformations may restore homoscedasticity [53].

As the program neither estimates the parameters of the distributions of the measurand, nor calculates any confidence intervals, it is not intended to analyze samples of measurements, but to be used as an educational and research tool, to explore and analyze the relation between diagnostic accuracy measures and measurement uncertainty.

All major general or medical statistical software packages (Matlab^®^, NCSS^®^, R, SAS^®^, SPSS^®^, Stata^®^ and MedCalc^®^) include routines for the calculation and plotting of various diagnostic accuracy measures and their confidence intervals. The program presented in this work provides 269 different types of plots of diagnostic accuracy measures (Figure 2), many of which are novel. To the best of our knowledge, not one of the abovementioned programs or any other software provides this range of plots without advanced statistical programming.

## 5. Conclusions

The program developed for this work clearly demonstrates various aspects of the relation between diagnostic accuracy measures and measurement uncertainty and can be used as a flexible, user-friendly, interactive educational or research tool in medical decision-making, to explore and analyze this relation.

## Figures and Tables

**Table 1:** A 2 × 2 contingency table.

		Populations	
		Non-Diseased	Diseased
**Test Results**	**Negative**	true negative (*TN*)	false negative (*FN*)
	**Positive**	false positive (*FP*)	true positive (*TP*)

**Table 2:** Natural frequency and probability definitions of diagnostic accuracy measures.

Measure	Natural Frequency Definition	Probability Definition
Sensitivity (*Se*)	$\frac{TP}{FN+TP}$	Pr(T|D)
Specificity (*Sp*)	$\frac{TN}{TN+FP}$	$Pr\left(\bar{T}|\bar{D}\right)$
Positive Predictive Value (*PPV*)	$\frac{TP}{FP+TP}$	Pr(D|T)
Negative Predictive Value (*NPV*)	$\frac{TN}{TN+FN}$	$Pr\left(\bar{D}|\bar{T}\right)$
Overall Diagnostic Accuracy (*ODA*)	$\frac{TN+TP}{TN+FN+TP+FP}$	$Pr\left(D\right)\;Pr\left(T|D\right)+Pr\left(\bar{D}\right)\;\mathrm{Pr}\left(\bar{T}|\bar{D}\right)$
Diagnostic Odds Ratio (*DOR*)	$\frac{TN\;TP}{FN\;FP}$	$\frac{\frac{Pr\left(T|D\right)}{Pr\left(\bar{T}|D\right)}}{\frac{Pr\left(T|\bar{D}\right)}{Pr\left(\bar{T}|\bar{D}\right)}}$
Likelihood Ratio for a Positive Result (*LR*+)	$\frac{TP\left(FP+TN\right)}{FP\left(FN+TP\right)}$	$\frac{Pr\left(T|D\right)}{Pr\left(T|\bar{D}\right)}$
Likelihood Ratio for a Positive Result (*LR−*)	$\frac{FN\left(FP+TN\right)}{TN\left(FN+TP\right)}$	$\frac{Pr\left(\bar{T}|D\right)}{Pr\left(\bar{T}|\bar{D}\right)}$
Youden’s Index (*J*)	$\frac{TN\;TP-FN\;FP}{\left(TN+FP\right)\left(FN+TP\right)}$	$Pr\left(T|D\right)+Pr\left(\bar{T}|\bar{D}\right)-1$
Euclidean Distance (*ED*)	$\sqrt{{\left(\frac{FN}{FN+TP}\right)}^{2}+{\left(\frac{FP}{TN+FP}\right)}^{2}}$	$\sqrt{Pr{\left(\bar{T}|D\right)}^{2}+Pr{\left(T|\bar{D}\right)}^{2}}$
Concordance Probability (*CZ*)	$\frac{TN\;TP\;}{\left(TN+FP\right)\left(FN+TP\right)}$	$Pr\left(T|D\right)\;Pr\left(\bar{T}|\bar{D}\right)$
Risk (*R*)	$l_{0}+\frac{l_{TN}TN+l_{FN}FN+l_{TP}TP+l_{FP}FP}{TN+FN+TP+FP}$	$l_{0}+l_{TN}Pr\left(\bar{D}\right)\mathrm{Pr}\left(\bar{T}|\bar{D}\right)\;+l_{FN}Pr\left(D\right)Pr\left(\bar{T}|D\right)\;+l_{TP}Pr\left(D\right)Pr\left(T|D\right)\;+l_{FP}Pr\left(\bar{D}\right)Pr\left(T|\bar{D}\right)$

The symbols are explained in Appendix A.

**Table 3:** Definitions of diagnostic accuracy measures against sensitivity and specificity.

Measure	Definition
Positive Predictive Value (*PPV*)	$\frac{Se\;v}{Se\;v+\left(1-Sp\right)\left(1-v\right)}$
Negative Predictive Value (*NPV*)	$\frac{Sp\;\left(1-v\right)}{Sp\;\left(1-v\right)+\left(1-Se\right)v}$
Overall Diagnostic Accuracy (*ODA*)	Se v+Sp (1−v)
Diagnostic Odds Ratio (*DOR*)	$\frac{\frac{Se}{1-Se}}{\frac{1-Sp}{Sp}}$
Likelihood Ratio for a Positive Result (*LR*+)	$\frac{Se}{1-Sp}$
Likelihood Ratio for a Positive Result (*LR−*)	$\frac{1-Se}{Sp}$
Youden’s Index (*J*)	Se+Sp−1
Euclidean Distance (*ED*)	$\sqrt{{\left(1-Se\right)}^{2}+{\left(1-Sp\right)}^{2}}$
Concordance Probability (*CZ*)	Se Sp
Risk (*R*)	$\begin{array}{cc} l_{0}+l_{TN}Sp\;\left(1-v\right) & +l_{FN}\left(1-Se\right)v+l_{TP}Se\;v\\ & +l_{FP}\left(1-Sp\right)\left(1-v\right) \end{array}$

The symbols are explained in Appendix A.

**Table 4:** The parameter settings of Figure 12, Figure 13, Figure 14, Figure 15, Figure 16 and Figure 17 and Table 5.

Settings	Figure 12	Figure 13	Figure 14	Figure 15	Figure 16	Figure 17	Table 5
$\mu_{D}$	2.99	2.99	2.99	2.99	2.99	2.99	2.99
$\sigma_{D}$	0.75	0.75	0.75	0.75	0.75	0.75	0.75
$\mu_{\bar{D}}$	0.0	0.0	0.0	0.0	0.0	0.0	0.0
$\sigma_{\bar{D}}$	1.0	1.0	1.0	1.0	1.0	1.0	1.0
v	0.067	0.067	−	0.067	0.067	0.067	0.067
d	2.26	2.26	2.26	−	−	−	
$u_{a}$	−	−	0.023	0.023	0.023	0.023	0.023
$u_{b}$	−	−	0.23	0.23	0.23	0.23	0.23
$l_{0}$	−	−	1	−	1	1	1
$l_{TN}$	−	−	0	−	0	0	0
$l_{FN}$	−	−	100	−	100	100	100
$l_{TP}$	−	−	0	−	0	0	0
$l_{FP}$	−	−	76	−	76	76	76

The symbols of the settings column are explained in Appendix A.

**Table 5:** Optimal diagnostic thresholds.

			Optimal Diagnostic Threshold		
			First Test	Second Test	Relative Difference
**Optimizing DAM**	**Youden’s index**	*J*	1.637	1.623	0.009
	**Euclidean distance**	*ED*	1.676	1.663	0.008
	**concordance probability**	*CZ*	1.640	1.627	0.008
	**Risk**	*R*	2.258	2.290	−0.014

The optimal diagnostic thresholds with the respective parameters in Table 4**.**

## Supplementary Materials

Supplementary materials can be found at https://www.mdpi.com/2075-4418/10/9/610/s1.

## Appendix A

**Notation**

1. *Populations*

$\bar{D}$: nondiseased population

*D*: diseased population

2. *Test Outcomes*

$\bar{T}$: negative test result

*T:* positive test result

*TN:* true negative test result

*TP:* true positive test result

*FN*: false negative test result

*FP*: false positive test result

3. *Diagnostic Accuracy Measures*

*Se:* sensitivity

*Sp*: specificity

*PPV:* positive predictive value

*NPV*: negative predictive value

*ODA:* overall diagnostic accuracy

*DOR:* diagnostic odds ratio

*LR* +: likelihood ratio for a positive test result

*LR* −: likelihood ratio for a negative test result

*J:* Youden’s index

*ED:* Euclidean distance of a *ROC* curve point from the point (0,1)

*CZ:* concordance probability

*R:* risk

*ROC:* receiver operating characteristic curve

*AUC*: area under the *ROC* curve

*AOC:* area over the *ROC* curve

*PROC:* predictive receiver operating characteristic curve

4. *Parameters*

$\mu_{P}$: mean of the measurand of a single test in the population *P*

$\sigma_{P}$: standard deviation of the measurand of a single test in the population *P*

*v**:* prevalence of the disease

*d:* diagnostic threshold of a single test

*u:* standard measurement uncertainty of a single test

5. *Expected Loss*

$l_{0}$: expected loss for the testing procedure

$l_{TN}$: expected loss for a true negative result

$l_{FN}$: expected loss for a false negative result

$l_{TP}$: expected loss for a true positive result

$l_{FP}$: expected loss for a false positive result

6. *Functions and Relations*

*se* (*d,..*.): sensitivity function of a single test against its diagnostic threshold *d*

*sp* (*d,..*.): specificity function of a single test against its diagnostic threshold *d*

$se_{sp}\left(z,\ldots \right)$: sensitivity function of a single test against its specificity *z*

$sp_{se}\left(y,\ldots \right)$: specificity function of a single test against its sensitivity *y*

roc(…): receiver operator characteristic function of a screening or diagnostic test

auc(…): function of the area under the receiver operator characteristic curve

aoc(…): function of the area over the receiver operator characteristic curve

proc(…): predictive receiver operator characteristic relation of a screening or diagnostic test

ed(t,…): Euclidean distance function of the *ROC* curve point (t, roc(t,…)) from the point (0, 1)

Φ(x): cumulative distribution function of the standard normal distribution, evaluated at *x*

Ψ(x,μ,σ): cumulative distribution function of a normal distribution with mean *μ* and standard deviation *σ*, evaluated at *x*

S(x,μ,σ): survival function of a normal distribution with mean *μ* and standard deviation *σ*, evaluated at *x*

erf(x): error function, evaluated at *x*

erfc(x): complementary error function, evaluated at *x*

*Pr* (*a*): probability of an event *a*

*Pr*(*a*|*b*): probability of an event *a* given the event *b*

$F^{-1}\left(\ldots \right)$: The inverse function *F*

## Appendix B

**Software Availability and Requirements**

**Program name:** Relation

**Available at:** https://www.hcsl.com/Tools/Relation.nb

**Operating systems:** Microsoft Windows, Linux, Apple iOS

Programming language: Wolfram Language

**Other software requirements:** Wolfram Player^®^, freely available at: https://www.wolfram.com/player/ or Wolfram Mathematica^®^

**System requirements:** Intel^®^ Pentium™ Dual-Core or equivalent CPU and 2GB of RAM

**License:** Attribution - NonCommercial - ShareAlike 4.0 International Creative Commons License

## References

[1] Šimundić A.-M. (2009). Measures of diagnostic accuracy: Basic definitions. EJIFCC.

[2] Shiu S.-Y., Gatsonis C. (2008). The predictive receiver operating characteristic curve for the joint assessment of the positive and negative predictive values. Philos. Trans. A Math. Phys. Eng. Sci..

[3] McNeil B.J., Hanley J.A. (1984). Statistical approaches to the analysis of receiver operating characteristic (ROC) curves. Med. Decis. Mak..

[4] Hanley J.A., McNeil B.J. (1982). The meaning and use of the area under a receiver operating characteristic (ROC) curve. Radiology.

[5] Hilden J. (1991). The area under the ROC curve and its competitors. Med. Decis. Mak..

[6] Hatjimihail A.T. The Area Over a Receiver Operating Characteristic (ROC) Curve as an Index of Diagnostic Inaccuracy: Wolfram Demonstrations Project. 2011. (updated 3/7/2011). https://demonstrations.wolfram.com/TheAreaOverAReceiverOperatingCharacteristicROCCurveAsAnIndex/.

[7] Youden W.J. (1950). Index for rating diagnostic tests. Cancer.

[8] Hajian-Tilaki K. (2018). The choice of methods in determining the optimal cut-off value for quantitative diagnostic test evaluation. Stat. Methods Med. Res..

[9] Liu X. (2012). Classification accuracy and cut point selection. Stat. Med..

[10] Joint Committee for Guides in Metrology (2008). Evaluation of Measurement Data—Guide to the Expression of Uncertainty in Measurement.

[11] Kallner A., Boyd J.C., Duewer D.L., Giroud C., Hatjimihail A.T., Klee G.G., Lo S.F., Pennello G., Sogin D., Tholen D.W. (2012). Expression of Measurement Uncertainty in Laboratory Medicine; Approved Guideline.

[12] White G.H. (2008). Basics of estimating measurement uncertainty. Clin. Biochem. Rev..

[13] Oosterhuis W.P., Theodorsson E. (2016). Total error vs. measurement uncertainty: Revolution or evolution?. Clin. Chem. Lab. Med..

[14] Smith A.F., Shinkins B., Hall P.S., Hulme C.T., Messenger M.P. (2019). Toward a framework for outcome-based analytical performance specifications: A methodology review of indirect methods for evaluating the impact of measurement uncertainty on clinical outcomes. Clin. Chem..

[15] Ceriotti F., Fernandez-Calle P., Klee G.G., Nordin G., Sandberg S., Streichert T., Vives-Corrons J.-L., Panteghini M. (2017). Criteria for assigning laboratory measurands to models for analytical performance specifications defined in the 1st EFLM Strategic Conference. Clin. Chem. Lab. Med..

[16] Bloch D.A. (1997). Comparing two diagnostic tests against the same “Gold Standard” in the same sample. Biometrics.

[17] Sakia R.M. (1992). The box-cox transformation technique: A review. J. R. Stat. Soc. Ser. D (Statistician).

[18] Gillard J. (2012). A generalised Box–Cox transformation for the parametric estimation of clinical reference intervals. J. Appl. Stat..

[19] Wolfram S. (2017). An Elementary Introduction to the Wolfram Language.

[20] Wolfram Research (2019). I. Mathematica, Version 12.0..

[21] Hatjimihail A.T. Receiver Operating Characteristic Curves and Uncertainty of Measurement: Wolfram Demonstrations Project. 2007. (updated 6/12/2007). https://demonstrations.wolfram.com/ReceiverOperatingCharacteristicCurvesAndUncertaintyOfMeasure/.

[22] Hatjimihail A.T. Uncertainty of Measurement and Areas Over and Under the ROC Curves: Wolfram Demonstrations Project. 2009. (updated 4/20/2009). https://demonstrations.wolfram.com/UncertaintyOfMeasurementAndAreasOverAndUnderTheROCCurves/.

[23] Hatjimihail A.T. Uncertainty of Measurement and Diagnostic Accuracy Measures: Wolfram Demonstrations Project. 2009 (updated 5/26/2009). https://demonstrations.wolfram.com/UncertaintyOfMeasurementAndDiagnosticAccuracyMeasures/.

[24] Chatzimichail T. Analysis of Diagnostic Accuracy Measures: Wolfram Demonstrations Project. 2015. (updated 7/24/2015). https://demonstrations.wolfram.com/AnalysisOfDiagnosticAccuracyMeasures/.

[25] Chatzimichail T. Calculator for Diagnostic Accuracy Measures: Wolfram Demonstrations Project. 2018. (updated 4/25/2018). https://demonstrations.wolfram.com/CalculatorForDiagnosticAccuracyMeasures/.

[26] Chatzimichail T. Correlation of Positive and Negative Predictive Values of Diagnostic Tests: Wolfram Demonstrations Project. 2018. (updated 4/5/2018). https://demonstrations.wolfram.com/CorrelationOfPositiveAndNegativePredictiveValuesOfDiagnostic/.

[27] Chatzimichail T., Hatjimihail A.T. Calculation of Diagnostic Accuracy Measures: Wolfram Demonstrations Project. 2018. (updated 6/22/2018). https://demonstrations.wolfram.com/CalculatorForDiagnosticAccuracyMeasures/.

[28] Lim T.O., Bakri R., Morad Z., Hamid M.A. (2002). Bimodality in blood glucose distribution: Is it universal?. Diabetes Care.

[29] American Diabetes A (2019). 2. Classification and diagnosis of diabetes: Standards of medical care in diabetes-2019. Diabetes Care.

[30] Kupchak P., Wu A.H.B., Ghani F., Newby L.K., Ohman E.M., Christenson R.H. (2006). Influence of imprecision on ROC curve analysis for cardiac markers. Clin. Chem..

[31] Kroll M.H., Biswas B., Budd J.R., Durham P., Gorman R.T., Gwise T.E., Pharmd A.-B.H., Hatjimihail A.T., Hilden J., Song K. (2011). Assessment of the Diagnostic Accuracy of Laboratory Tests Using Receiver Operating Characteristic Curves; Approved Guideline.

[32] Lippi G., Simundic A.-M., Plebani M. (2020). Potential preanalytical and analytical vulnerabilities in the laboratory diagnosis of coronavirus disease 2019 (COVID-19). Clin. Chem. Lab. Med..

[33] Tang Y.-W., Schmitz J.E., Persing D.H., Stratton C.W. (2020). The laboratory diagnosis of COVID-19 Infection: Current issues and challenges. J. Clin. Microbiol..

[34] Deeks J.J., Dinnes J., Takwoingi Y., Davenport C., Leeflang M.M.G., Spijker R., Hooft L., van den Bruel A., Emperador D., Dittrich S. (2020). Diagnosis of SARS-CoV-2 infection and COVID-19: Accuracy of signs and symptoms; molecular, antigen and antibody tests; and routine laboratory markers. Cochrane Database Syst. Rev..

[35] Infantino M., Grossi V., Lari B., Bambi R., Perri A., Manneschi M., Terenzi G., Liotti I., Ciotta G., Taddei C. (2020). Diagnostic accuracy of an automated chemiluminescent immunoassay for anti-SARS-CoV-2 IgM and IgG antibodies: An Italian experience. J. Med. Virol..

[36] Mahase E. (2020). Covid-19: “Unacceptable” that antibody test claims cannot be scrutinised, say experts. BMJ.

[37] Kontou P.I., Braliou G.G., Dimou N.L., Nikolopoulos G., Bagos P.G. (2020). Antibody tests in detecting SARS-CoV-2 infection: A meta-analysis. Diagnostics (Basel).

[38] Theodorsson E. (2017). Uncertainty in measurement and total error: Tools for coping with diagnostic uncertainty. Clin. Lab. Med..

[39] Padoan A., Sciacovelli L., Aita A., Antonelli G., Plebani M. (2018). Measurement uncertainty in laboratory reports: A tool for improving the interpretation of test results. Clin. Biochem..

[40] Aggarwal R. (2018). Risk, complexity, decision making and patient care. JAMA Surg..

[41] Hatjimihail A.T. (2009). Estimation of the optimal statistical quality control sampling time intervals using a residual risk measure. PLoS ONE.

[42] Collins J., Albert P.S. (2016). Estimating diagnostic accuracy without a gold standard: A continued controversy. J. Biopharm. Stat..

[43] Zhou X.-H. (2011). Statistical Methods in Diagnostic Medicine.

[44] Atkinson A.B. (2020). The box-cox transformation: Review and extensions. Stat. Sci..

[45] Box G.E.P., Cox D.R. (1964). An analysis of transformations. J. R. Stat. Soc. Ser. B Stat. Methodol..

[46] Solberg H.E. (1987). Approved recommendation (1987) on the theory of reference values. Part 5. Statistical treatment of collected reference values. Determination of reference limits. Clin. Chim. Acta.

[47] Pavlov I.Y., Wilson A.R., Delgado J.C. (2012). Reference interval computation: Which method (not) to choose?. Clin. Chim. Acta.

[48] Sikaris K. (2012). Application of the stockholm hierarchy to defining the quality of reference intervals and clinical decision limits. Clin. Biochem. Rev..

[49] Daly C.H., Liu X., Grey V.L., Hamid J.S. (2013). A systematic review of statistical methods used in constructing pediatric reference intervals. Clin. Biochem..

[50] Ozarda Y., Sikaris K., Streichert T., Macri J., IFCC Committee on Reference Intervals and Decision Limits (C-RIDL) (2018). Distinguishing reference intervals and clinical decision limits—A review by the IFCC Committee on Reference Intervals and Decision Limits. Crit. Rev. Clin. Lab. Sci..

[51] Wilson J.M.G., Jungner G. (1968). Principles and Practice of Screening for Disease.

[52] Petersen P.H., Horder M. (1992). 2.3 Clinical test evaluation. Unimodal and bimodal approaches. Scand. J. Clin. Lab. Investig..

[53] Analytical Methods Committee AN (2019). Why do we need the uncertainty factor?. Anal. Methods.

