# Supplementary material for: A Software Tool for Exploring the Relation between Diagnostic Accuracy and Measurement Uncertainty
Source: Diagnostics (Basel). 2020 Aug 19;10(9):610. doi: 10.3390/diagnostics10090610 (PMC7555914; doi:10.3390/diagnostics10090610)
Supplement: Supplementary file 1 [file diagnostics-10-00610-s001.pdf]

Relation

A Software Tool for Exploring the Relation between Diagnostic Accuracy and Measurement Uncertainty

Theodora Chatzimichail, MD [a], ***Aristides T. Hatjimihail, MD, PhD*** [b]

Hellenic Complex Systems Laboratory, Kostis Palamas 21, Drama 66131, Greece, [a]tc@hcsl.com, [b]ath@hcsl.com

INTERFACE

1.1 ABOUT THE PROGRAM CONTROLS

The numerical settings are defined by the user with menus or sliders. Sliders can be finely manipulated by holding down the *alt* key or *opt* key while dragging the mouse. They be even more finely manipulated by also holding the *shift* and/or *ctrl* keys.

Dragging with the mouse rotates the three-dimensional plots, while dragging with the mouse while pressing the *ctrl*, *alt*, or *opt* keys zooms in or out.

1.2 RANGE OF THE INPUT PARAMETERS

v: 0.00000001 – 0.99

$\mu_D, \mu_{\overline{D}}$: 0 - 6

$\sigma_D, \sigma_{\overline{D}}$: 0.01 - 6

d: Minimum of $(\mu_D - 3.5\sigma_D, \mu_{\overline{D}} - 3.5\sigma_{\overline{D}})-$ Maximum of $(\mu_D + 3.5\sigma_D, \mu_{\overline{D}} + 3.5\sigma_{\overline{D}})$

u : 0 - 6

l_0: 0 - 1000

l_{TN}: 0 - 1000

l_{FN}: 0 - 1000

l_{TP}: 0 - 1000

l_{FP}: 0 – 1000

Iinitial plot points per axis: 10 – 50

1.3 RANGE OF THE COORDINATES OF THE PLOTS

There are two options for the range of coordinates to be included in each plot:

1) *Full*: All the calculated coordinate points are plotted.
2) *Partial*: The distribution of coordinate values is found and any points sufficiently far out in the distribution are not considered.

1.4 INPUT AND OUTPOUT

The program provides in five modules and six submodules plots and tables of diagnostic accuracy measures of two screening or diagnostic tests for a single measurand, differing in measurement uncertainty, applied at a single point in time in a diseased and a non-diseased population.

Singularity points are excluded from the plots.

Indeterminate results of the calculation modules represent numerical quantities whose magnitudes cannot be determined, because they are either too small or too large.

1.4.1 ROC curves

Input

The user defines:

1) The prevalence of the disease.
2) The means and the standard deviations of the measurand in the diseased and non-diseased populations.
3) The standard measurement uncertainty of each test.

Output

1) a) The plots of the *ROC* curves of the two tests and
   b) A table with the areas under and over the *ROC* curves (*AUC* and *AOC* respectively), or
2) The plots of the *PROC* curves of the two tests.

1.4.2 Diagnostic accuracy measures plots

The module presents in three submodules plots of diagnostic accuracy measures of the tests.

1.4.2.1 Diagnostic accuracy measures versus diagnostic threshold

Input

1) The user defines:
   a) The prevalence of the disease.
   b) The means and the standard deviations of the measurand in the diseased and non-diseased populations.
   c) The standard measurement uncertainty of each test.
   d) The expected loss for:
      i) Testing,

ii) A true positive result,
iii) A true negative result,
iv) A false positive result and
v) A false negative result.

2) The diagnostic accuracy measure function to be plotted, by selecting:
   a) The measure:
      i) Sensitivity (Se),
      ii) Specificity (Sp),
      iii) Overall diagnostic accuracy (ODA),
      iv) Positive predictive value (PPV),
      v) Negative predictive value (NPV),
      vi) Diagnostic odds ratio (DOR),
      vii) Likelihood ratio for a positive test result (LR +),
      viii) Likelihood ratio for a negative test result (LR -),
      ix) Youden's index (J),
      x) Euclidean distance (ED),
      xi) Concordance probability (CZ),
      xii) Risk (R).
   b) The codomain of the function, that is
      i) The value of the measure,
      ii) The partial derivative of the measure with respect to standard measurement uncertainty,
      iii) The difference between the measures of the two tests,
      iv) The relative difference between the measures of the two tests,
      v) The ratio of the measures of the two tests.
   c) The range of the coordinate points to be plotted:
      i) Full,
      ii) Partial.

Output

Plots of:

1) The values of the measure of each test,
2) The partial derivatives of the measure of each test with respect to standard measurement uncertainty,
3) The difference between the measures of the two tests,
4) The relative difference between the measures of the two tests,
5) The ratio of the measures of the two tests,

versus diagnostic threshold (d).

1.4.2.2 Diagnostic accuracy measures versus prevalence

Input

The user defines:

1) The means and the standard deviations of the measurand in the diseased and non-diseased populations.
2) The diagnostic threshold.
3) The standard measurement uncertainty of each test.
4) The expected loss for:
   a) Testing,
   b) A true positive result,
   c) A true negative result,
   d) A false positive result and
   e) A false negative result.
2) The diagnostic accuracy measure function to be plotted, by selecting:
   a) The measure:
      i) Sensitivity (Se),
      ii) Specificity (Sp),
      iii) Overall diagnostic accuracy (ODA),
      iv) Positive predictive value (PPV),
      v) Negative predictive value (NPV),
      vi) Diagnostic odds ratio (DOR),
      vii) Likelihood ratio for a positive test result (LR +),
      viii) Likelihood ratio for a negative test result (LR -),
      ix) Youden's index (J),
      x) Euclidean distance (ED),
      xi) Concordance probability (CZ),
      xii) Risk (R).
   b) The codomain of the function, that is
      i) The value of the measure,
      ii) The partial derivative of the measure with respect to standard measurement uncertainty,
      iii) The difference between the measures of the two tests,
      iv) The relative difference between the measures of the two tests,
      v) The ratio of the measures of the two tests.
3) The range of the coordinate points to be plotted:
   a) Full,
   b) Partial.

Output

Plots of:

1) The values of the measure of each test,
2) The partial derivatives of the measure of each test with respect to standard measurement uncertainty,
3) The difference between the measures of the two tests,
4) The relative difference between the measures of the two tests,
5) The ratio of the measures of the two tests,

versus prevalence (v).

1.4.2.3 *Diagnostic accuracy measures versus uncertainty*

Input

The user defines:

1) The prevalence of the disease.
2) The means and the standard deviations of the measurand in the diseased and non-diseased populations.
3) The diagnostic threshold.
4) The expected loss for:
   i) Testing,
   ii) A true positive result,
   iii) A true negative result,
   iv) A false positive result and
   v) A false negative result.
2) The diagnostic accuracy measure function to be plotted:
   a) Sensitivity (*Se*),
   b) Specificity (*Sp*),
   c) Overall diagnostic accuracy (*ODA*),
   d) Positive predictive value (*PPV*),
   e) Negative predictive value (*NPV*),
   f) Diagnostic odds ratio (*DOR*),
   g) Likelihood ratio for a positive test result (*LR* +),
   h) Likelihood ratio for a negative test result (*LR* -),
   i) Youden's index (*J*),
   j) Euclidean distance (*ED*),
   k) Concordance probability (*CZ*),
   l) Risk (*R*).
3) The range of the coordinate points to be plotted:
   i) Full,
   ii) Partial.

Output

Plots of the values of the measure of each test versus uncertainty (u).

1.4.3 Diagnostic accuracy measures relations plots

The module presents in three submodules plots of the relations between diagnostic accuracy measures of the tests.

1.4.3.1 *Diagnostic accuracy measures versus sensitivity or specificity*

Input

The user defines:

1) The prevalence of the disease

2) The means and the standard deviations of the measurand in the diseased and non-diseased populations.
3) The standard measurement uncertainty of each test.
4) The expected loss for:
   a) Testing,
   b) A true positive result,
   c) A true negative result,
   d) A false positive result and
   e) A false negative result.
2) The diagnostic accuracy measure function to be plotted by selecting:
   a) The measure:
      i) Overall diagnostic accuracy (ODA),
      ii) Positive predictive value (PPV),
      iii) Negative predictive value (NPV),
      iv) Diagnostic odds ratio (DOR),
      v) Likelihood ratio for a positive test result (LR +),
      vi) Likelihood ratio for a negative test result (LR -),
      vii) Youden's index (J),
      viii) Euclidean distance (ED),
      ix) Concordance probability (CZ),
      x) Risk (R).
   b) The domain of the function:
      i) The sensitivity (Se) of the test,
      ii) The specificity (Sp) of the test.
   c) The codomain of the function, that is
      i) The value of the measure,
      ii) The partial derivative of the measure with respect to standard measurement uncertainty,
      iii) The difference between the measures of the two tests,
      iv) The relative difference between the measures of the two tests,
      v) The ratio of the measures of the two tests.
3) The range of the coordinate points to be plotted:
   a) Full,
   b) Partial.

Output

Plots of:

1) The values of the measure of each test,
2) The partial derivatives of the measure of each test with respect to standard measurement uncertainty,
3) The difference between the measures of the two tests,
4) The relative difference between the measures of the two tests,
5) The ratio of the measures of the two tests,

versus sensitivity (*Se*) or specificity (*Sp*).

1.4.3.2 The diagnostic accuracy measures versus sensitivity and specificity

Input

The user defines:

1) The prevalence of the disease.
2) The means and the standard deviations of the measurand in the diseased and non-diseased populations.
3) The standard measurement uncertainty of each test.
4) The expected loss for:
   c) Testing,
   d) A true positive result,
   e) A true negative result,
   f) A false positive result and
   g) A false negative result.
4) The diagnostic accuracy measure function to be plotted by selecting:
   a) The measure:
      i) Overall diagnostic accuracy (ODA),
      ii) Positive predictive value (PPV),
      iii) Negative predictive value (NPV),
      iv) Diagnostic odds ratio (DOR),
      v) Likelihood ratio for a positive test result (LR +),
      vi) Likelihood ratio for a negative test result (LR -),
      vii) Youden's index (J),
      viii) Euclidean distance (ED),
      ix) Concordance probability (CZ),
      x) Risk (R).
   b) The codomain of the function, that is
      i) The value of the measure,
      ii) The partial derivative of the measure with respect to standard measurement uncertainty.
5) The range of the coordinate points to be plotted:
   a) Full,
   b) Partial.

Output

Three-dimensional line plots of the values or the derivatives of the measure of each test versus its sensitivity (*Se*) and specificity (*Sp*).

1.4.3.3 Diagnostic accuracy measures relations

Input

The user defines:

1) The prevalence of the disease.

2) The means and the standard deviations of the measurand in the diseased and non-diseased populations.
3) The standard measurement uncertainty of each test.
4) The expected loss for:
   c) Testing,
   d) A true positive result,
   e) A true negative result,
   f) A false positive result and
   g) A false negative result.
5) The relation betweenthe diagnostic accuracy measures to be plotted, by selecting any two of the following:
   a) Overall diagnostic accuracy (*ODA*),
   b) Positive predictive value (*PPV*),
   c) Negative predictive value (*NPV*),
   d) Diagnostic odds ratio (*DOR*),
   e) Likelihood ratio for a positive test result (*LR* +),
   f) Likelihood ratio for a negative test result (*LR* -),
   g) Youden's index (*J*),
   h) Euclidean distance (*ED*),
   i) Concordance probability (*CZ*),
   j) Risk (*R*).
6) The range of the coordinate points to be plotted:
   a) Full,
   b) Partial.

Output

Plots of the respective parametric equations of any two of the above diagnostic accuracy measures.

1.4.4 Diagnostic accuracy measures calculator

Input

The user defines:

1) The prevalence of the disease.
2) The means and the standard deviations of the measurand in the diseased and non-diseased populations.
3) The diagnostic threshold.
4) The standard measurement uncertainty of each test.
5) The expected loss for:
   a) Testing,
   b) A true positive result,
   c) A true negative result,
   d) A false positive result and
   e) A false negative result,.

Output

A table of the values and the relative differences of the following diagnostic accuracy measures of the two tests for the selected diagnostic threshold:

b) Sensitivity (*Se*),
c) Specificity (*Sp*),
d) Overall diagnostic accuracy (*ODA*),
e) Positive predictive value (*PPV*),
f) Negative predictive value (*NPV*),
g) Diagnostic odds ratio (*DOR*),
h) Likelihood ratio for a positive test result (*LR* +),
i) Likelihood ratio for a negative test result (*LR* -),
j) Youden's index (*J*),
k) Euclidean distance (*ED*),
l) Concordance probability (*CZ*),
m) Risk (*R*).

1.4.5 Optimal diagnostic accuracy measures calculator

Input

The user defines:

1) The prevalence of the disease.
2) The means and the standard deviations of the measurand in the diseased and non-diseased populations.
3) The diagnostic threshold.
4) The expected loss for:
    a) Testing,
    b) A true positive result,
    c) A true negative result,
    d) A false positive result and
    e) A false negative result.
5) The objective or loss function to find the diagnostic threshold for the following optima:
    a) Maximum Youden's index (*J*),
    b) Minimum Euclidean distance (*ED*),
    c) Maximum concordance probability (*CZ*),
    d) Minimum risk (*R*).

Output

1) The optimal diagnostic thresholds for the two tests.
2) A table of the values of the following diagnostic accuracy measures of the two tests at the respective optimal diagnostic thresholds and of their relative differences:
    a) Sensitivity (*Se*),
    b) Specificity (*Sp*),
    c) Overall diagnostic accuracy (*ODA*),

d) Positive predictive value (*PPV*),
e) Negative predictive value (*NPV*),
f) Diagnostic odds ratio (*DOR*),
g) Likelihood ratio for a positive test result (*LR* +),
h) Likelihood ratio for a negative test result (*LR* -),
i) Youden's index (*J*),
j) Euclidean distance (*ED*),
k) Concordance probability (*CZ*),
l) Risk (*R*).
